# Development and Validation of an LC-MS/MS Method for Simultaneous Determination of Short Peptide-Based Drugs in Human Blood Plasma

**DOI:** 10.3390/molecules27227831

**Published:** 2022-11-14

**Authors:** Elizaveta N. Fisher, Evgeny S. Melnikov, Vladimir Gegeckori, Natalya V. Potoldykova, Dmitry V. Enikeev, Kirill A. Pavlenko, Snezana Agatonovic-Kustrin, David W. Morton, Galina V. Ramenskaya

**Affiliations:** 1 I.M. Sechenov First MSMU of the Ministry of Health of the Russian Federation, Sechenov University, 8, Trubetskaya Street, 119991 Moscow, Russia; 2 LLC «CPHA», 20/3, Nauchny Proezd, 117246 Moscow, Russia; 3 Clinical Hospital. I. V. Davidovsky, Department of Health of the City of Moscow, 11, Yauzskaya Street, 119027 Moscow, Russia; 4 Institute for Urology and Reproductive Health, I.M. Sechenov First MSMU of the Ministry of Health of the Russian Federation, Sechenov University, 8, Trubetskaya Street, 119991 Moscow, Russia; 5 Moscow Clinical Scientific Center, 86, Shosse Enthuziastov, 111123 Moscow, Russia; 6 Department of Pharmacy and Biological Sciences, La Trobe University, Edwards Road, Flora Hill, VIC 3550, Australia

**Keywords:** LC-ESI-MS/MS, peptide drugs, GnRH analogs, somatostatin analogue, solid-phase extraction

## Abstract

A novel HPLC-ESI-MS/MS method for simultaneous gonadotropin-releasing hormone (GnRH) analogs and somatostatin analog quantitation was developed and validated. The developed method was successfully applied to pharmacokinetic studies. The sample preparation process included solid-phase extraction (SPE). Effective chromatographic separation of the analytes and internal standard (dalargin) was achieved with a C18 column, using a gradient elution with two mobile phases: 0.1% *v*/*v* formic acid (aqueous solution) and 0.1% *v*/*v* formic acid (acetonitrile solution). The linearity of the method was demonstrated within a concentration range of 0.5–20 ng/mL, with correlation coefficients between 0.998–0.999 for goserelin, buserelin, triptorelin, and octreotide, respectively. The relative standard deviation (RSD, %) values for method accuracy and precision did not exceed 20% at the lower level of quantitation (LLOQ) or 15% at other concentration levels.

## 1. Introduction

Gonadotropin-releasing hormone (GnRH) is a decapeptide released by hypothalamic neurons, which stimulates GnRH-mediated pituitary secretion of both luteinizing hormone (LH) and follicle-stimulating hormone (FSH). With a short half-life (several minutes), GnRH is secreted directly to the pituitary blood vessels; therefore, the GnRH level is not measured in peripheral blood [1,2]. Goserelin, buserelin, and triptorelin are GnRH agonists, a relatively new class of drugs. The long-term administration of these agonists results in a significant decrease in estrogen and testosterone blood levels. They downregulate the sex glands’ activity due to pituitary desensitization during constant GnRH receptor stimulation. GnRH analogs are the first line of drugs for the treatment of hormone-dependent cancers, such as prostate and breast cancer [3,4]. 

GnRH analogs are a modified version of a naturally occurring GnRH, decapeptides with a structure similar to the native hormone [5], but with a greater affinity to the GnRH receptors, a prolonged half-life, and a greater resistance to enzymatic breakdown compared to native GnRH. Endogenous GnRH has low protein-binding affinity, and GnRH analogs usually form drug–protein complexes during the distribution phase due to the presence of hydrophobic groups in their structure [6,7]. Amino acid substitution in synthetic GnRH analogs results in their higher affinity to the GnRH receptors, longer half-life (up to 2 h), and higher potency (approximately 10 times that of native GnRH) [8,9]. 

Octreotide is another example of a long-acting synthetic analog of polypeptide hormone somatostatin used in clinical practice. Octreotide is an octapeptide in which the four-amino-acid sequence of somatostatin is retained. It inhibits the release of various peptides in the gastropancreatic endocrine system [10,11,12]. It has more specific, more potent, and longer-acting inhibitory effects than somatostatin. It is used to prevent postoperative complications after pancreatic resection and as a surgical approach to the treatment of pancreatic adenocarcinoma, benign tumor, and chronic pancreatitis. For pancreatic cancer, as well as for other epithelial tumors, the currently available treatments (e.g., chemo- or radiotherapy) are only of limited efficacy [13]. Octreotide, being a somatostatin analog, requires close clinical monitoring because of the numerous physiological actions of somatostatin in the body.

Immunochemical assays [14,15,16], capillary zone electrophoresis [17,18,19,20,21], and liquid chromatography [22,23,24,25] are the main methods for the quantitation of peptide-based therapeutics in biological fluids. These methods consider the molecular structure of peptide-based drugs and their physical and chemical properties. Immunochemical assays (enzyme-linked immunosorbent assays (ELISAs) and radioimmunoassays (RIAs), being relatively simple methods, usually demonstrate high sensitivity (the measurement of pg/mL levels of the analyte) [26,27]. However, immunochemical methods have several limitations, such as lack of selectivity and cross-reactivity with other peptides, which usually lead to false positives. Moreover, the use of RIA methods can be hazardous for laboratory personnel due to the use of radiolabeled molecules [28,29,30].

Chromatography and capillary zone electrophoresis, combined with UV-detection, have several limitations when used for the analysis of biological fluids. In particular, the analyte concentrations should be relatively high in order to reduce matrix interference. Unfortunately, peptide blood concentrations are usually quite low [31,32]. Therefore, the most preferable method for peptide-based drugs’ quantitation is to use liquid chromatography–electrospray ionization tandem mass spectrometry (LC-ESI-MS/MS). Detection is performed in the positive ion mode, as peptides are polar molecules, and they can easily become protonated. Moreover, the HPLC-MS/MS method is highly selective, accurate, rapid, and safe. 

The main aim of this research was to develop and validate a novel method for the quantification of short peptide-based drugs that can be readily adapted to clinical pharmacokinetic studies. 

According to previous studies [33,34,35,36,37], solid-phase extraction is the method of choice for sample preparation, leading to lower values of LLOQ. An alternative method is protein precipitation, in which acetonitrile and 10% trichloroacetic acid solution are used as precipitating reagents [38]. The use of acetonitrile led to an incomplete recovery of the analyte, and a 10% trichloroacetic acid solution ensured the extraction of the analyte only when working with high concentrations (at the level of μg/mL).

In this work, for the first time, a standardized method for the quantitative determination of peptides in human blood plasma by HPLC-MS/MS was developed and validated. The method was applied to the simultaneous quantification of gonadotropin-releasing hormone (GnRH) analogs, goserelin, buserelin, and triptorelin and quantification of octreotide in human blood plasma.

## 2. Results and Discussion

### 2.1. Method Validation

The developed method was validated according to the guidelines of the FDA (U.S. Food and Drug Administration) [39] and the EMA (European Medicines Agency) [40] for selectivity, linearity, matrix effect, accuracy (intra-day, inter-day), precision (intra-day, inter-day), lower limit of quantification (LLOQ), carryover, and stability.

#### 2.1.1. Selectivity

**Figure 1:**
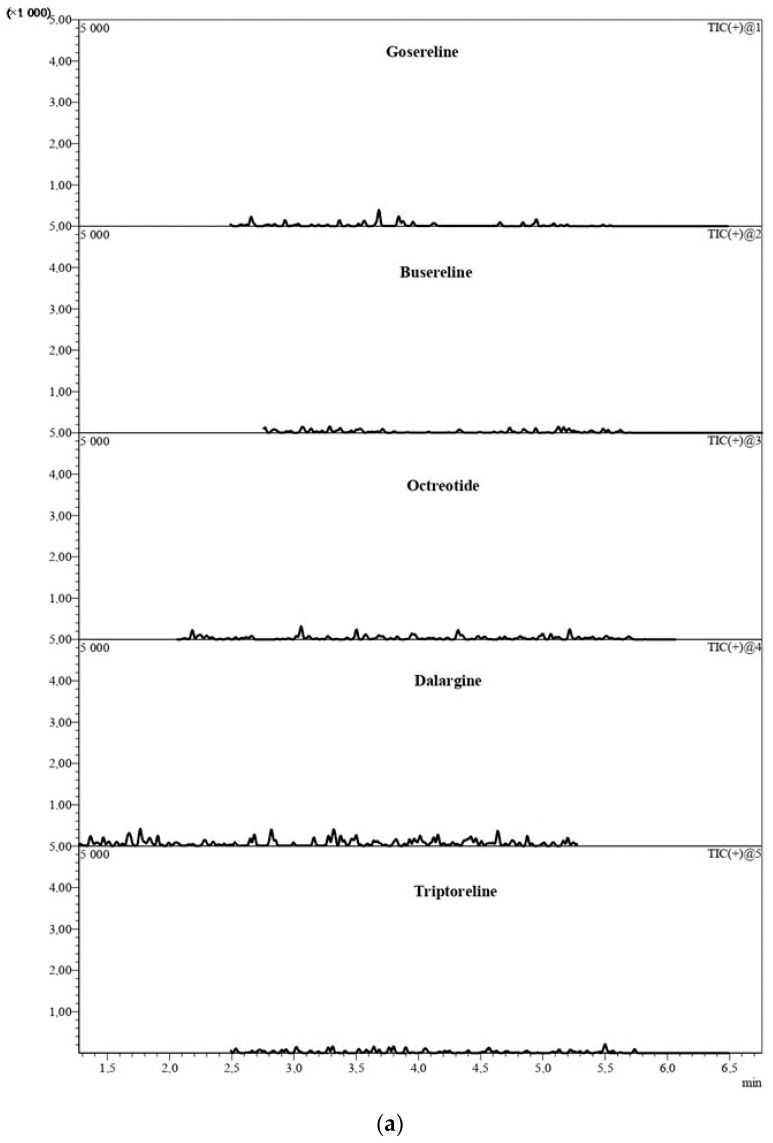

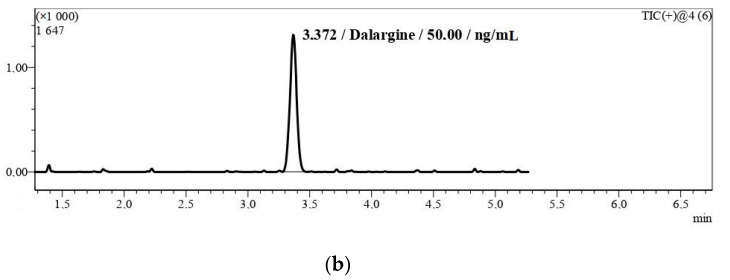
The chromatograms of (**a**) blank plasma samples and (**b**) blank plasma samples with IS.

Method selectivity was determined by comparing the chromatograms of six blank plasma samples, obtained from different sources, with standard-spiked plasma samples. During analysis, the samples were spiked with different analyte concentrations at a range of 0.5–20 ng/mL, each with an IS concentration of 50 ng/mL. The chromatograms of blank plasma samples (Figure 1a) and the chromatogram of the blank plasma sample with IS (Figure 1b) showed no interfering peaks and multiple reaction-monitoring (MRM) transitions at the retention time, similar to those of each analyte and the IS.

The obtained values of the correlation coefficients (*R*) were considered valid (≥0.99). The calculated values of the absolute relative error (*E*, %) for calibration sample concentrations are presented in Table 1.

#### 2.1.2. Accuracy and Precision

To assess method accuracy and precision, blank plasma samples spiked with each analyte’s working standard solution at the concentrations of 0.5 ng/mL, 1 ng/mL, 10 ng/mL, and 20 ng/mL were analyzed five times at each of the four analyte concentration levels within one run (intra-day precision). Inter-day accuracy and precision were assessed within three consecutive days. Accuracy was assessed quantitatively by using relative error: to describe accuracy, the relative error was calculated, and precision was described as the relative standard deviation (Table 2).

#### 2.1.3. Matrix Effect

To determine the biological matrix effect in quantitative goserelin, buserelin, triptorelin, and octreotide analysis, the matrix effect (ME, %) was calculated. The matrix effect was determined as the peak areas of each analyte added to the blank and extracted, divided by the peak areas of peptides for the corresponding neat solution (matrix-free). Further, during method validation, the IS-normalized matrix effect was calculated as analyte ME percentage divided by the IS ME percentage. Recovery (RE, in percent) was calculated as the ratio of analyte peak area in the pre-extraction and the post-extraction spiked samples. Recovery and matrix effect were assessed at concentrations of 1 ng/mL and 20 ng/mL. The results are presented in Table 3.

During ME, % assessment, the lowest values at the concentration level of 1 ng/mL were observed for octreotide (66%) and triptorelin (73%), and for other peptides, the ME, % values were higher than 85%. The ME, % values at the concentration level of 20 ng/mL varied from 79% to 89%. The lowest recovery at the 1 ng/mL concentration level was determined for triptorelin (53%) and at the 20 ng/mL concentration level for triptorelin (65%) and octreotide (65%). The other peptides have demonstrated recovery values of 69% to 82% (for the 1 ng/mL concentration level) and 77% to 88% (for the 20 ng/mL concentration level).

#### 2.1.4. Lower Limit of Quantification (LLOQ)

The LLOQ was determined based on the linearity, accuracy, and precision data. The LLOQ for the method was determined as the minimum analyte (goserelin, buserelin, or dalargin) plasma concentration within the linear range that can be determined with acceptable accuracy and precision (less than 20%). The valid LLOQ concentration was determined to be 0.5 ng/mL.

#### 2.1.5. Stability

The concentration measured after storage for each analyte was within 15% of the nominal concentration in all tested conditions.

#### 2.1.6. Carryover

There were no interfering peaks at the retention time for goserelin, buserelin, triptorelin, and the IS. Thus, there was no carryover observed.

### 2.2. Practical Application of the Developed Methodology 

**Figure 2:**
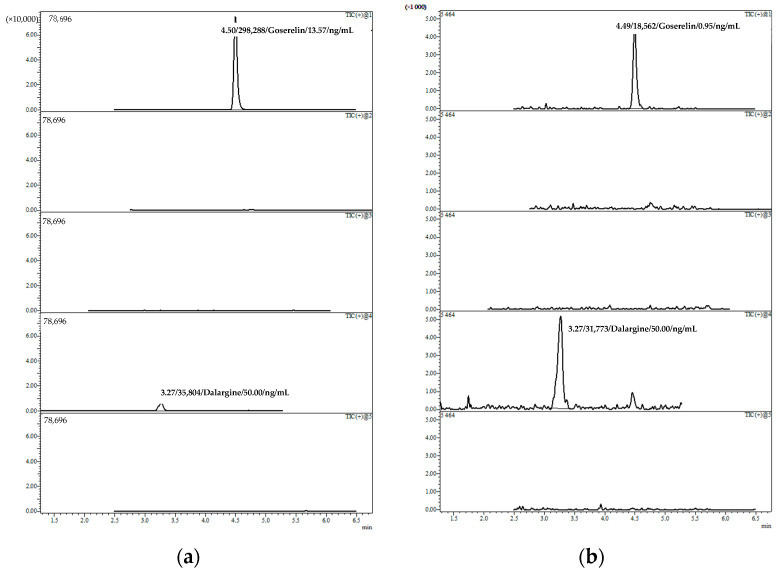

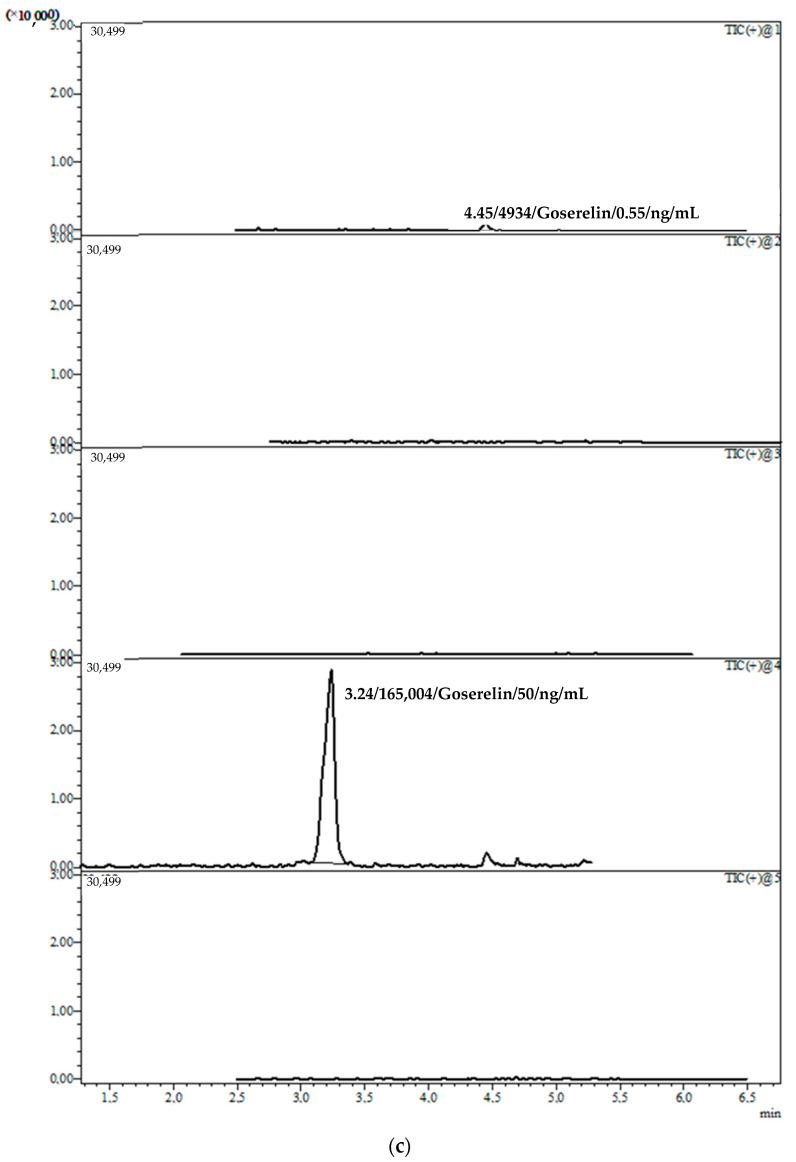
Representative chromatograms of the patient’s plasma sample for (**a**) patient No. 1; (**b**) patient No. 2, and (**c**) patient No. 3.

The developed method for anti-cancer peptide-based therapeutics blood plasma quantitation was successfully applied to therapeutic monitoring in patients with prostate cancer. The patients received goserelin, triptorelin, or buserelin drugs at different stages of the therapy. The findings are presented in Table 4. Chromatograms of the patients’ plasma samples are presented in Figure 2.

Table 4 shows that most of the patients received goserelin, one patient received triptorelin (patient No. 5), and one patient received buserelin (patient No. 9) as a therapeutic substitution for goserelin during continuous anti-cancer treatment. Patients No. 1 (therapy duration is about 6 months), No. 7 (therapy duration is about 1 year), and No. 8 (therapy duration is more than 2 years) demonstrated the highest goserelin plasma concentrations of 13.57 ng/mL, 7.81 ng/mL, and 16.23 ng/mL, respectively. Patients No. 2 (therapy duration is 9 days) and No. 3 (therapy duration is about 1 month) showed goserelin concentrations at the LLOQ level: 0.95 ng/mL and 0.55 ng/mL, respectively. Goserelin concentrations in patient Nos. 4, 5, 6, and 9’s plasma samples were lower than the LLOQ. Moreover, sample collection was performed either for several days after a subcutaneous injection or at the end of the treatment course, as GnRH analogs’ concentration tends to increase on the 12th day after goserelin (3.6 mg) or buserelin (3.75 mg) injection and on the 3rd–4th day after goserelin (10.8 mg) or triptorelin (11.25 mg) administration. The obtained data demonstrated that goserelin plasma concentration starts to increase during the second week of therapy. Thus, to perform the pharmacokinetic study, it is preferable to collect patients’ samples only on the 10th–12th day after drug administration, when there is a peak or plateau concentration of the drug in blood plasma.

## 3. Materials and Methods

### 3.1. Chemicals and Reagents

Goserelin acetate, buserelin acetate, and formic acid (extra pure, ≥98%) were purchased from Sigma-Aldrich (Saint Louis, MO, USA). Triptorelin acetate was purchased from TLC Pharmaceutical Standards Ltd. (Newmarket, ON, Canada). Dalargin diacetate was obtained from American Custom Chemicals Corporation, USA. Methanol (HPLC grade) was purchased from Panreac-Quimica SA (Barcelona, Spain). Zoladex^®^ was from AstraZeneca (Macclesfield, UK). Acetonitrile (LC-MS grade) was obtained from Biosolve B.V. (Valkenswaard, The Netherlands). Ultrapure water was obtained from a Milli-Q Integral A10, Millipore water purification system (Millipore, Guyancourt, France).

### 3.2. Preparation of Stock and Working Standard Solutions

Stock solutions (50 μg/mL) were prepared by dissolving 10.5 mg of goserelin acetate, buserelin acetate, or triptorelin acetate (equal to 10.0 mg of free base) in acetonitrile/deionized water (1:1, *v*/*v*) to a total volume of 200.0 mL. Working standard solutions of each peptide were then prepared by diluting the stock solution with an acetonitrile/deionized water solution (1:1, *v*/*v*), to obtain solutions in the concentration range of 0.5–20 ng/mL (i.e., 0.5, 1, 2.5, 5, 10, and 20 ng/mL). The internal standard (IS) solution of dalargin diacetate had a final concentration of 50 μg/mL. All prepared standard solutions were stored at 2–8 °C.

The stability of peptides was evaluated under different handling and storage conditions at two concentration levels (1 ng/mL and 20 ng/mL), in standard solutions (stock and working) and in matrix samples. Freeze–thaw (three cycles) and short-term (for 24 h) and long-term (in deep freezer) stability were assessed during method validation. 

### 3.3. Calibration and Quality Control Samples

Linearity was assessed over the concentration range of 0.5–20.0 ng/mL for each analyte (goserelin, buserelin, and triptorelin). To assess the method linearity, six blank plasma samples were spiked with suitable working standard solutions to obtain calibration samples with analyte concentrations of 0.5 ng/mL, 1.0 ng/mL, 2.5 ng/mL, 5.0 ng/mL, 10.0 ng/mL, and 20.0 ng/mL, each with an IS concentration of 50.0 ng/mL. Calibration curves were fitted by plotting the ratio of analyte to IS peak area versus the ratio of analyte to IS nominal concentrations in blood plasma samples. The correlation coefficients (*R*) for goserelin, buserelin, triptorelin and octreotide were 0.998, 0.999, 0.998, and 0.998, respectively.

Calibration samples were prepared by spiking 990 μL of blank plasma with 10 μL of the suitable working standard solution. Quality control samples (QC) were prepared in the same manner, by spiking 990 μL of blank plasma with 10 μL of working standard solution (0.5, 1, 10, and 20 ng/mL). All of the prepared samples were stored between −75 °C to −80 °C.

### 3.4. Sample Preparation

A 1000 μL volume of blank plasma was placed in a 2 mL Eppendorf tube. Then, 10 μL of internal standard solution (50 μg/mL) was added to the tube, and the mixture was vortexed. An amount of 100 μL of the resulting sample was then transferred to another centrifuge tube, and 200 μL of methanol/deionized water/formic acid (60:40:0.08) mixture was added, followed by 500 μL of methanol. The sample was then centrifuged at 14,500 rpm for 15 min. 

For the solid-phase extraction (SPE), the SPE cartridge was pre-treated with 1 mL methanol, followed by 1 mL of deionized water. The sample mixture was diluted with 500 μL of deionized water and gently loaded onto an SPE Oasis^®^ HLB 1cc 30 mg cartridge (Waters Corporation, Milford, MA, USA). The SPE cartridge was washed with 1 mL deionized water, followed by 1 mL of methanol/deionized water (60:40) mixture. The sample was then eluted with 1 mL 0.1% formic acid (methanol solution) and evaporated to dryness (for 20 min) at 45 °C under a stream of nitrogen. The dry residue was then reconstituted with 100 μL of methanol/deionized water/formic acid (60:40:0.08) mixture.

### 3.5. Analytical Instrumentation

The LC-MS/MS analysis was performed with a Nexera X2 system (Shimadzu, Japan) and a LCMS-8040 Shimadzu tandem mass spectrometer (Shimadzu, Japan) equipped with a pump, an automatic thermostatic column oven, and a thermostatic autosampler. LabSolutions software (Shimadzu, Japan) was used to control the HPLC and mass spectrometer and to process the data. 

### 3.6. LC-MS/MS Operation Conditions

The chromatographic separation was performed on a Jupiter^®^ C18 column (5 μm, 50 × 4.6 mm 300 Å) (Phenomonex, Torrance, CA, USA). The mobile phase consisted of 0.1% *v*/*v* formic acid aqueous solution (solvent A) and 0.1% *v*/*v* formic acid in acetonitrile (solvent B). The mobile phase composition was changed gradually in a gradient elution (Table 5) with a constant flow rate of 1.2 mL/min. The temperature of the column oven was kept at 30 °C. After the sample preparation procedure, 30 μL of supernatant was injected into the LC-MS/MS system.

### 3.7. Development and Optimization of the MS/MS Parameters

**Figure 3:**
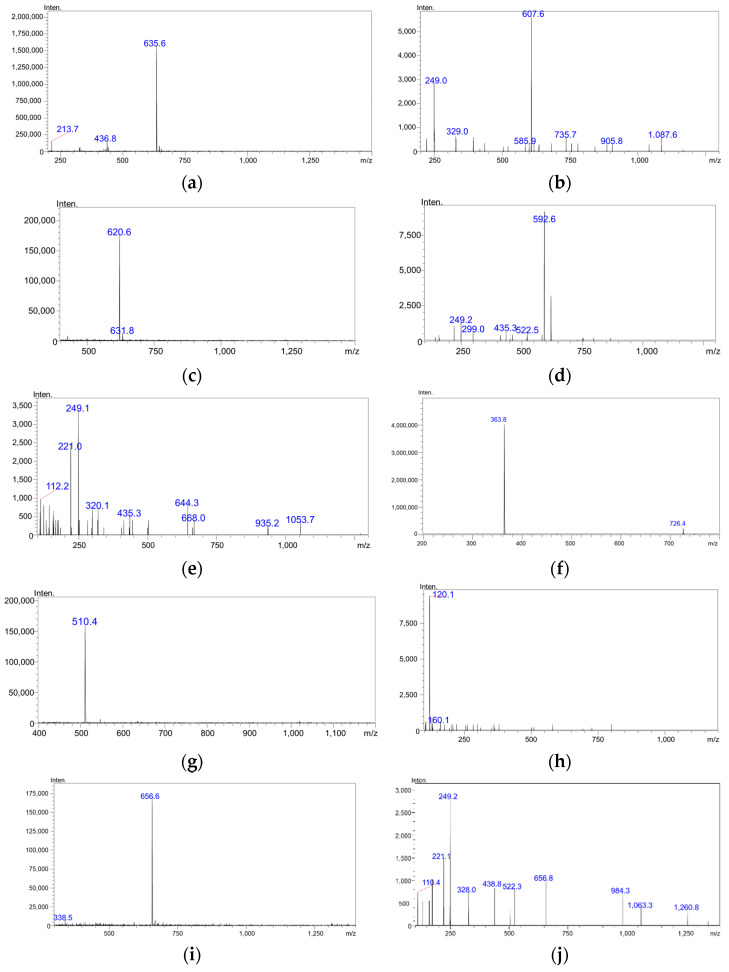
Current mass spectrum produced by LC-ESI-MS/MS for (**a**) goserelin total ion; (**b**) goserelin product ion *m*/*z* 635.60, positive ESI, collision energy −20 V; (**c**) buserelin total ion; (**d**) buserelin product ion *m*/*z* 592.60 mass spectra, positive ESI, collision energy −19 V, (**e**) buserelin product ion *m*/*z* 241.10 mass spectra, positive ESI, collision energy −37 V, (**f**) dalargin total ion; (**g**) octreotide total ion; (**h**) octreotide product ion *m*/*z* 120.10 mass spectra, positive ESI, collision energy −34 V; (**i**) triptorelin total ion; (**j**) triptorelin product ion *m*/*z* 249.20 mass spectra, positive ESI, collision energy −40 V.

Under the positive ESI conditions, goserelin produced a predominantly protonated molecule [M + 2H]^2+^ at *m*/*z* 635.60 (Figure 3a), buserelin produced a protonated molecule [M + 2H]^2+^ at m/z 620.60 (Figure 3c), triptorelin produced a protonated molecule [M + 2H]^2+^ at *m*/*z* 656.50 (Figure 3j), and octreotide produced a protonated molecule [M + 2H]^2+^ at *m*/*z* 510.40 (Figure 3h) in full-scan mass spectra. All four peptides showed intense double-protonated molecular ions. These ions were chosen as the precursor ions for a further search of product ions in order to develop a MRM mode analysis. Formic acid, the dynamic modifier for the mobile phase, was shown to improve the analyte ionization efficacy. Thus, a mobile phase consisting of 0.1% *v*/*v* formic acid aqueous solution (solvent A) and 0.1% *v*/*v* formic acid acetonitrile solution (solvent B) was used for further method development and validation. Product ions produced after precursor ion fragmentation were then analyzed during the MS/MS optimization process. Fragment ions at *m*/*z* 607.60 (for goserelin), *m*/*z* 249.10, and 592.0 (for buserelin) and *m*/*z* 248.95 (for triptorelin) and *m*/*z* 120.05 (for octreotide) showed the highest intensity in mass spectra. The mass spectra for each analyte are given in Figure 3.

Detection was performed with triple quadruple tandem mass spectrometry in the positive ion mode (5 kW) using the MRM mode. Nitrogen was used as a nebulizer (3 L/h) and drying (20 L/h) gas. The desolvation temperature was set at 200 °C, and the source temperature was 400 °C. Detection conditions are summarized in Table 6.

## 4. Conclusions

The developed and validated LC-MS/MS method, which used SPE in the sample preparation procedure, enabled therapeutic concentrations of goserelin to be determined up to 0.5 ng/mL. The analytical range of the developed method can be considered valid, as it is within expected concentration values in patients. The obtained results confirm that the developed method can be used in clinical pharmacokinetic studies in patients with prostate cancer. It can determine optimal time points for sample collection in order to obtain samples with drug concentrations within the therapeutic range. Moreover, the validated method can also be applied to the bioequivalence studies of generic drug products.

## Figures and Tables

**Table 1:** Summary of linearity data for goserelin, buserelin, triptorelin, and octreotide over the concentration range of 0.5–20.0 ng/mL.

Analyte	Regression Analysis	Nominal Concentration (ng/mL)	Measured Concentration (ng/mL)	Maximum *E* (%)
Goserelin	*y* = 1.9602*x* + 0.0007 (*R* = 0.998)	0.50	0.46–0.58	16.0
		1.00	0.96–1.14	14.0
		2.50	2.34–2.72	8.8
		5.00	4.64–5.23	7.2
		10.00	9.37–10.47	6.3
		20.00	17.49–21.04	12.5
Buserelin	*y* = 8.2778*x* + 0.0006 (*R* = 0.999)	0.50	0.48–0.53	6.00
		1.00	0.91–1.03	9.00
		2.50	2.13–2.17	14.8
		5.00	5.11–5.34	6.8
		10.00	10.84–11.22	12.20
		20.00	19.75–20.12	1.25
Triptorelin	*y* = 0.8506*x* + 0.0014 (*R* = 0.998)	0.50	0.48–0.53	6.00
		1.00	0.91–1.03	9.00
		2.50	2.13–2.17	14.8
		5.00	5.11–5.34	6.8
		10.00	10.84–11.22	12.20
		20.00	19.75–20.12	1.25
Octreotide	*y* = 1.6853*x* + 0.0003 (*R* = 0.998)	0.50	0.49–0.53	6.0
		1.00	1.00–1.12	12.0
		2.50	2.46–2.70	8.00
		5.00	4.89–5.14	2.80
		10.00	10.17–10.39	3.90
		20.00	17.82–19.16	10.90

The calculated values of relative standard deviation (RSD, %) and relative error (*E*, %) meet the guideline acceptance criteria (less than 20% for LLOQ level and less than 15% for other concentration levels).

**Table 2:** Intra-day and inter-day accuracy and precision.

		Intra-Day (*n* = 5)			Inter-Day (*n* = 10)		
Analyte	Nominal Concentration/ng/mL	Average Measured Concentration/ng/mL	RSD/%	*E*/%	Average Measured Concentration/ng/mL	RSD/%	*E*/%
Goserelin	0.50	0.46	8.71	7.60	0.51	8.40	1.00
	1.00	1.04	11.10	3.80	1.02	6.18	2.10
	10.00	9.73	7.26	2.70	10.31	6.10	3.07
	20.00	19.49	7.63	2.54	20.21	7.03	1.07
Buserelin	0.50	0.51	8.89	2.40	0.48	4.67	4.60
	1.00	0.97	6.60	2.80	0.96	7.27	4.50
	10.00	10.44	4.75	4.36	9.99	3.99	0.06
	20.00	20.44	5.80	2.18	20.07	5.31	0.33
Triptorelin	0.50	0.50	6.15	0.80	0.49	7.88	1.40
	1.00	1.00	7.67	0.40	1.01	8.04	0.90
	10.00	10.14	8.28	1.36	9.81	6.99	1.92
	20.00	19.93	10.14	0.34	19.79	8.02	1.03
Octreotide	0.50	0.49	7.79	1.20	0.49	5.29	2.20
	1.00	0.98	8.86	2.40	1.03	5.84	2.50
	10.00	10.17	10.17	1.74	10.25	6.58	2.48
	20.00	19.98	4.11	0.09	19.75	2.28	1.27

All the results were within the acceptable limits according to the guidelines’ requirements.

**Table 3:** The matrix effect (ME) and recovery (RE) for each analyte.

Substance	Matrix Effect (ME)/%		IS-Normalized Matrix Effect (ME_norm_)/%		Recovery (RE)/%	
	1 ng/mL	20 ng/mL	1 ng/mL	20 ng/mL	1 ng/mL	20 ng/mL
Goserelin	91	89	107	101	77	87
Buserelin	87	81	102	92	82	77
Triptorelin	73	79	85	90	53	65
Octreotide	66	79	77	89	69	65
	50 ng/mL					
IS	85	88	-	-	80	88

**Table 4:** Goserelin, buserelin, and triptorelin quantitation in patients’ blood plasma samples.

Patient No.	Received Drug, Active Substance, Dose	Duration of the Received Therapy	Day of the Sample Collection	Measured Concentration/ng/mL
1	Zoladex^®^, goserelin acetate, 10.8 mg	6 months	29th/84	13.57
2	Zoladex^®^, goserelin acetate, 3.6 mg	9 days	8th/28	0.95
3	Zoladex^®^, goserelin acetate, 10.8 mg	2 months	59th/84	0.55
4	Zoladex^®^, goserelin acetate, 10.8 mg	3 days	3rd/84	-
5	Triptorelin-long^®^, triptorelin acetate, 11.25 mg	3 months	83rd/84	-
6	Zoladex^®^, goserelin acetate, 10.8 mg	3 months	74th/84	-
7	Zoladex^®^, goserelin acetate, 10.8 mg	8 months	10th/84	7.81
8	Zoladex^®^, goserelin acetate, 3.6 mg	2 years	16th/28	16.23
9	Buserelin-depo^®^, buserelin acetate, 3.75 mg	2 years	27th/28	-

**Table 5:** Gradient elution program.

Time/Minutes	Solvent B, %
0–0.3	5
0.3–4.6	5–35
4.6–5.1	35–95
5.1–6.5	95
6.5–8.2	95–5
8.2–10	5

**Table 6:** Mass spectrometry detection parameters (MRM mode).

Analyte	RT/Min	Precursor Ion/*m*/*z*	Product Ion/*m*/*z*	Collision Energy/V
Goserelin	4.61	635.50	607.45	−20.0
Buserelin	4.88	620.45	248.90	−37.0
			592.35	−19.0
Triptorelin	4.59	510.40	120.05	−34.0
Dalargin	3.37	363.85	120.10	−43.0
			135.95	−22.0
			492.30	−13.0

Total analysis time for a single injection was 10 min. The retention time for goserelin, buserelin, triptorelin, and dalargin (IS) was around 4.61 min, 4.88 min, 4.59 min, and 3.37 min, respectively.

## Data Availability

The data presented in this study are available on request from the corresponding author.
